# Hydroxyapatite–bioglass nanocomposites: Structural, mechanical, and biological aspects

**DOI:** 10.3762/bjnano.13.123

**Published:** 2022-12-12

**Authors:** Olga Shikimaka, Mihaela Bivol, Bogdan A Sava, Marius Dumitru, Christu Tardei, Beatrice G Sbarcea, Daria Grabco, Constantin Pyrtsac, Daria Topal, Andrian Prisacaru, Vitalie Cobzac, Viorel Nacu

**Affiliations:** 1 Institute of Applied Physics, 5 Academiei str., MD-2028, Chisinau, Republic of Moldova; 2 National Institute for Laser, Plasma and Radiation Physics, Laser Department, 409th Atomistilor str., RO-77125, Magurele, Bucharest, Romania; 3 University Politehnica Bucharest, 313 Splaiul Independentei, Bucharest, 060042, Romania; 4 National Institute for R&D in Electrical Engineering ICPE-CA, 313 Splaiul Unirii, 031066, Bucharest, Romania; 5 Nicolae Testemitanu State University of Medicine and Pharmacy, 165 Stefan cel Mare si Sfant ave., MD-2004, Chisinau, Republic of Moldova

**Keywords:** bioactivity, hardness, microstructure, nanocomposites, porosity

## Abstract

This research work focuses on the fabrication and study of a series of nanocomposites consisting of two types of hydroxyapatite (HA), obtained by precipitate (HAP) and sol–gel (HAG) methods, and a boro-silico-phosphate bioglass. The microstructure and chemical, mechanical, and biological properties as functions of three factors, namely (i) the type of hydroxyapatite, (ii) glass content, and (iii) sintering temperature, were investigated. It was found that all of these factors affect the final composition and microstructure, especially, porosity, which shows significantly lower values for HAP-based composites than for HAG-based ones and higher values for higher glass content. This, in turn, has an impact on the microhardness, which exhibits a strong correlation with porosity, as well as on the mineralization capability and cell viability due to the different dissolution rate.

## Introduction

Within the last decades increasing emphasis is placed on the development of new synthetic biomaterials that are perfectly compatible with biological tissue and provide special functionalities for healing, regeneration, and substitution of diseased or injured bones, as an alternative to “allograft” and “autograft” transplantology methods, which have a number of drawbacks. An ideal biomaterial to replace the bone tissue must integrate in the surrounding bone or soft tissue by stimulating osteoinduction and octeoconduction [1] at their interface in physiologic environment.

Synthetic hydroxyapatite (HA), Ca_10_(PO_4_)_6_(OH)_2_, although being very similar to the mineral composition of bone, has a low resorption in physiologic environment and, therefore, does not form a tough bond with the bone [2]. The development of hydroxyapatite–bioglass (HA-BG) composites aimed to overcome this problem [3–5]. In these composites, the biocompatibility of HA is combined with the bioactivity of the glass, which has a higher dissolution rate and promotes the formation of a carbonated hydroxyapatite (CHA) layer on its surface, which is responsible for implant–bone bonding [6].

The composition of the most famous bioglass, 45S5 Bioglass [7], includes the principal elements of the bone, Ca, P, and Na, in the form of oxides, and SiO_2_, which was proven to be a very useful component for CHA formation, as well as for angiogenesis processes and collagen formation [8–9]. Since the discovery of 45S5 Bioglass, a wide range of bioglasses have been elaborated and investigated, which can be divided into the following large groups: (i) silicate-based bioglasses (SBG), (ii) phosphate-based bioglasses (PBG), and (iii) borate-based bioglasses (BBG). The chemical composition of the glass, the ratio of its constituent oxides, and the glass structure have a determinative influence on its biological response in the physiologic environment [7,10–18]. The main role of SBG is the formation of CHA layer, whereas the role of PBG and BBG is the delivery of Ca and P ions, responsible for the osteogenesis, and other therapeutic elements due to their higher solubility. The addition of different dopants to the glass can enhance the osteogenesis (Mg, Sr, Fe, and Zr) or can yield particular properties such as angiogenesis (Cu and Co) and antimicrobial activity (Ag, Zn, Ce, Ga, and Cu). Also, dopants can change the glass solubility [13,15–19].

The presence of the glass in the HA-BG composites may stimulate the transformation of HA into tricalcium phosphate (TCP) during the sintering at high temperatures, inducing more reactivity of the material in the physiologic environment [4,20]. The HA-TCP transformation can be controlled through the glass fraction and the sintering temperature [21–23]. Moreover, the addition of the glass can contribute to the improvement of mechanical characteristics of the HA-BG composites, such as hardness, fracture toughness, and compressive strength, which is important for load-bearing applications [21–22,24–26]. Thus, BGs can induce new chemical, biological, and mechanical properties to HA-BG composites, which can be controlled by modifying glass fraction and composition.

In this study, we have elaborated and investigated HA-BG composites containing a boro-silico-phosphate bioglass of the following composition: 10B_2_O_3_-12MgO-8K_2_O-40P_2_O_5_-20SiO_2_-5ZnO-5CeO_2_ (mol %). This composition was chosen in order to combine high solubility of the glass with increased bioactivity, due to the high fraction of P_2_O_5_ and, simultaneously, the rather high fraction of SiO_2_. The inclusion of magnesium and potassium in conjunction with the high glass solubility will provide an enhanced delivery of these elements, which are important for bone formation [24,27–29]. Boron was shown to promote the formation of amorphous calcium phosphate, which is beneficial for normal metabolism and bone tissue repair; besides, the addition of B_2_O_3_ to HA inhibits the thermal decomposition of HA and significantly enhances bending strength and fracture toughness [30]. It was shown as well that B_2_O_3_ in the phosphate glass composition increases its hardness and fracture toughness [31]. The doping of bioglasses with Zn and Ce has two effects. On the one hand, it induces antibacterial activity, and, on the other hand, it promotes the formation and mineralization of bone tissue [19,32].

The HA-BG composites investigated in this work contained two types of HA, obtained either by the sol–gel method (HAG) or by the precipitation method (HAP). It was shown that HA resulting from the two methods significantly differs regarding the crystallite form and size, stoichiometry, surface activity, and Ca/P ratio [33–34]. Therefore, it was of interest to compare the influence of the two different types of HA on the overall behavior of the HA-BG composites. Moreover, two more modifying factors, namely BG fraction and sintering temperature, were introduced to study their influence on structure, mechanical properties, and biological activity depending on the type of HA (HAP or HAG) used for fabricating the HA-BG composites.

## Materials and Methods

### Preparation of composites

The composite samples were obtained from hydroxyapatite prepared by a precipitation method (HAP) or by a sol–gel method (HAG) combined with a bioglass of definite composition. For the preparation of HAP powder we used the method described in a previous work [35] with small modifications, according to the reaction


[1]

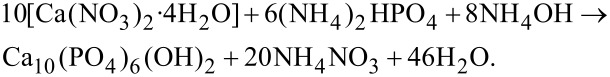



Raw materials obtained from Merck and Fluka of analytical purity were dosed corresponding to the stoichiometry of Ca_10_(PO_4_)_6_(OH)_2_, for which the Ca/P ratio is *r* = 1.67. The mixture was homogenized for 2 h at a temperature of 40–60 °C and pH 8–9, kept for 24 h at room temperature, filtered, and washed. Subsequently, the precipitate was dried in an oven at 80 °C for 24 h and then ground and calcined at 900 °C for 2 h. The nanoscale crystallite size of the obtained HAP was about 62 nm.

The HAG powder was obtained using Ca(NO_3_)_2_·4H_2_O and P_2_O_5_ (Merck) as precursors and ethanol as dispersion medium, all of analytical purity; the sol–gel method was described elsewhere [36] (method I - HAG 1b). After the calcination at 900 °C for 2 h, the samples were ground to obtain the HAG powder of about 55 nm particle size.

The bioglass composition is presented in Table 1. The samples were prepared by an unconventional wet route followed by melting [31,37–38]. As raw materials, the ultra-purity grade reagents boron oxide (B_2_O_3_), magnesium oxide (MgO,) potassium carbonate (K_2_CO_3_), phosphoric acid (H_3_PO_4_), silicon dioxide (SiO_2_), zinc oxide (ZnO), and cerium oxide (CeO_2_) have been used.

**Table 1:** Bioglass oxide composition.

Sample/oxide		B_2_O_3_	MgO	K_2_O	P_2_O_5_	SiO_2_	ZnO	CeO_2_	Total

BG	mol %	10	12	8	40	20	5	5	100
	wt %	6.91	4.8	7.47	56.32	11.92	4.04	8.54	100

The glass preparation comprised the following steps: (i) Preparation of raw materials, that is, the reagents were introduced in H_3_PO_4_ solution under continuous stirring. (ii) Drying of the mixture on an electrical heating plate at 130–140 °C. (iii) Thermal treatment of mixture, that is, the dried mixture was heated up to 240 °C in an electrical oven, than introduced in an alumina crucible and heated in an electric furnace in two steps, namely a pre-melting step at low heating rate of about 50 °C/h from 240 up to 800 °C, followed by a melting step, at a higher heating rate of 250 °C/h, up to the melting temperature of 1200 °C, where the mixture maintained for 0.5 h. To obtain a homogeneous glass, an alumina stirrer homogenized the melt at 200–240 rpm. (iv) Casting, that is, the melt was cast into graphite molds, previously preheated at 1.1 *T*_g_. (v) Cooling of the glass in air. (vi) Grinding the obtained bioglass in an agate mortar. (vii) Sieving of the BG powder to obtain powder of particle sizes below 64 μm.

The HA-BG composites were obtained by mixing 90 or 95 wt % of HA (HAP or HAG) powder with, respectively, 10 or 5 wt % of BG powder. An aqueous solution of 7% polyvinyl alcohol (PVA) was added to the mixture. The proportion of added PVA solution was 5% for 95% mixture. The mixture was than pressed in a metallic mold with cylindrical shape (12 mm diameter) using a hydraulic press at 1.5 or 2.0 tf for 60 s. The obtained samples were sintered according to the sintering program (Table 2), in order to eliminate the binder (PVA) and to densify the sanples, with four different final temperatures from 1100 to 1250 °C. The final samples were disc plates with a diameter of 10–12 mm and a thickness of about 3 mm. As reference samples, pure HAP or HAG ceramics without BG addition were prepared using the same processing procedure. The samples were labeled depending on the hydroxyapatite type used for their preparation (HAP or HAG), the percentage of BG (5% or 10%) and the final sintering temperatures, that is, “HAP-5BG-1200” is the HAP-based sample with 5% BG sintered at 1200 °C.

**Table 2:** Sintering program.

Temperature	Time [h]

*T*_amb_–250 °C	2
250 °C	0.5
250–650 °C	3
650 °C	0.5
650–1100/1150/1200/1250 °C	2
1100/1150/1200/1250 °C	3
1100/1150/1200/1250 °C–*T*_amb_	10

### Density and porosity measurements

The measurement method is the buoyancy technique, which utilizes Archimedes' principle: a body immersed in fluid indicates an apparent loss in weight equal to the weight of the fluid it displaces. In order to determine the porosity of the samples a preparation consisting of one hour boiling of the samples in water was made. The samples were weighted in air before boiling, in air after boiling, and in water after boiling with a precision of 0.0006 g.

The apparent density was calculated using the formula


[2] $$\rho_{\text{a}}=\frac{m_{0}\cdot \rho_{{\text{H}}_{2}\text{O}}}{m_{2}-m_{1}},$$

where ρ_a_ is the apparent density in g/cm^3^, *m*_0_ is the initial mass of the samples in g, *m*_2_ is the mass of the samples in air after boiling in g, and *m*_1_ is the mass of the samples in water after boiling in g.

The following formula was used for the calculation of the apparent porosity (open porosity), *P*_a_:


[3] $$P_{\text{a}}=\frac{m_{2}-m_{0}}{m_{2}-m_{1}}\cdot 100,$$

using the above annotations. The errors of measurement for density and porosity were ±0.05 g/cm^3^ and ±0.05%, respectively.

### X-ray diffraction

With the help of a Bruker D8 ADVANCE X-ray diffractometer, the evolution of the formation of hydroxyapatite and of the intermediate compounds was studied, on samples calcined at different temperatures, as well as after burning. The peaks recorded for 2θ in the range of 20–60˚ were then compared with the ICDD-PDF: 01-086-0740, ICDD-PDF: 01-073-4869, and ICDD-PDF: 00-009-0169 files. By processing the diffractograms, using the most representative peaks, the parameters of the elementary cell were calculated. Considering the main peak of each diffractogram with the highest intensity, the average size of the crystallite, *D_hkl_*, was calculated using the Scherrer equation:


[4] $$D_{hkl}=\frac{k\lambda }{\text{FWHM}\cos\theta },$$

where *k* is a dimensionless constant called the form factor, usually having a value of 0.9. λ is the X-ray wavelength, in this case λ = 1.54060 Å, FWHM (full width at half maximum) is the width at the half height of the peak, θ is the Bragg angle at which the reflection takes place, and *hkl* are the Miller indices associated with the crystallographic plane.

The crystallinity fraction was evaluated using the following formula:


[5] $$X_{\text{c}}=1-\frac{V_{112/300}}{I_{112/300}},$$

where *V*_112/300_ represents the intensity ratio between the peaks associated with the crystallographic planes (112) and (300) expressed in counts per second, and *I*_300_ represents the intensity of the characteristic peak of the crystallographic plane (300) expressed in counts per second.

### Scanning electron microscopy

To determine the microstructural characteristics, such as micromorphology of the obtained materials and the qualitative and quantitative distribution of the granular phase and pores, SEM analysis of the sintered materials was performed using a Zeiss Auriga FESEM-FIB electron microscope. For SEM analysis, the samples underwent a special cleaning process for 6 min in a nitrogen plasma jet in a FISCHONE Plasma Cleaner. A FEI QUANTA INSPECT S SEM was used to observe structure and morphology of the samples after their treatment in simulated body fluid, as well as to acquire the energy-dispersive X-ray (EDX) spectra and the elemental composition.

### Mechanical properties

The mechanical properties were investigated by means of depth-sensing microindentation (MI). Before the MI measurements, the samples were polished with sandpaper with grit sizes of P1000, P2000, and P3000, consecutively, and with wet Cr_2_O_3_ powder on a cloth for final refinement to obtain a mirror-like surface. The depth-sensing MI was carried out on a PMT-3NI-02 instrumented nano/microindentation device using a Vickers diamond tetrahedral pyramid as an indenter. For the calculation of microhardness (*H*), the Oliver–Pharr method was applied [39]. The range of used loads was 20, 40, 50, 100, and 200 mN, and at least ten indentations per each load were made for the calculation of *H*.

### Bioactivity investigation

For the bioactivity assessment of the samples, simulated body fluid (SBF) was prepared according to Kokubo and Takadama [40]. The as-prepared SBF was stored in a dry refrigerator at 5–10 °C, as indicated by Kokubo and Takadama. The samples were introduced in SBF for 3, 5, 10, 15 and 30 days. The SBF volume used for soaking the samples was calculated according to *V*_s_ = *S*_a_/10, where *V*_s_ is the volume of SBF (mL), and *S*_a_ is the apparent surface area of the specimen (mm^2^) [40]. The surface of the samples was then investigated by SEM to estimate their apatite-forming ability. The pH value of SBF was measured before and after the insertion of the sintered sample using a Multi-parameter analyzer Consort C1010, version 2.0, which has the possibility to measure pH, voltage (mV), and temperature (°C). The precision of the pH measurement was 10^−2^.

### MTT assay

The MTT assay was performed on mesenchymal stem cells (MSCs) previously isolated from six-months-old Wistar rat bone marrow, cultured in HiMesoXL™ mesenchymal stem cell expansion medium (HiMedia, India) and stored at −80 °C in FBS (Lonza, Belgium) with 10% DMSO (Alchimia, Italy). The ethical approval, as well as the protocol used for the isolation of the mesenchymal stem cells is described in the Annex. After thawing, cells from the third passage were cultured in DMEM/Ham F12 (Sigma, Germany) cell culture medium with 10% FBS and antibiotics/antimycotics in 75 cm^2^ flasks until 70–80% confluence.

The samples for the MTT tests were prepared in the form of discs of 3 mm diameter and 0.7 mm thickness. Before testing, the samples were sterilized in 70% alcohol for two hours and washed twice with HBSS (HiMedia, India). The test was performed in 24-well plates. A number of 1·10^4^ MSCs were introduced in each well and incubated at 37 °C in 5% CO_2_ humid environment. After 24 h, the samples were placed in the wells. Each sample was tested in three repetitions, namely after 24, 48, and 72 h. The 5% (v/v) MTT (Sigma, USA) working solution was prepared from 0.5% (w/v) MTT stock solution in DMEM/Ham F12 cell culture media. After removing the samples and cell culture media, MTT working solution was poured in the plates followed by incubation of the plates at 37 °C, 5% CO_2_ for 2 h, then the formazan crystals were dissolved in isopropanol (StanChem, Poland). The absorbance was measured at 570 nm with a spectrophotometer (Synergy H1, BioTek).

## Results and Discussions

### XRD analysis

**Figure 1:**
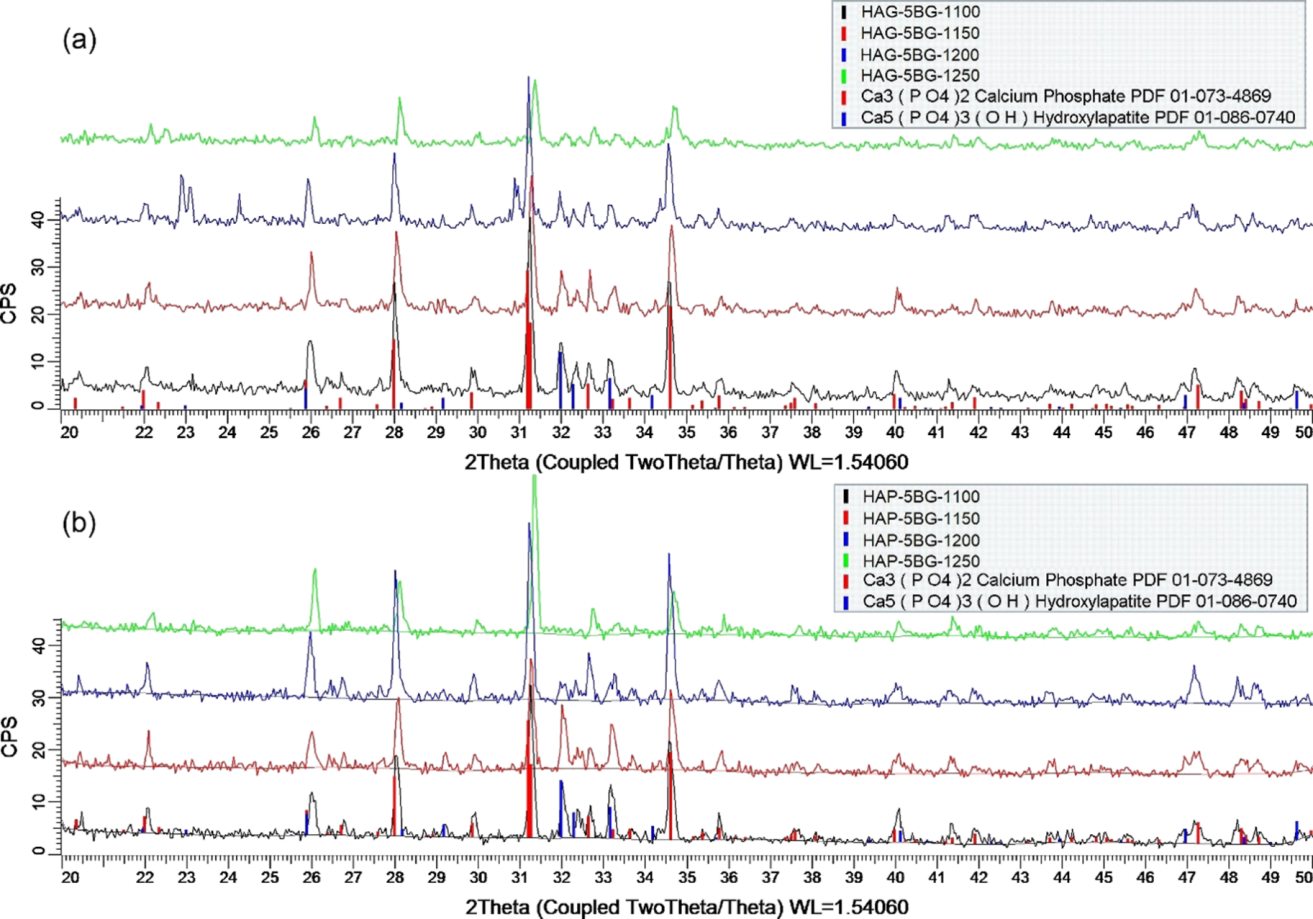
XRD patterns for (a) HAG-based composites and (b) HAP-based composites with 5% BG sintered at 1100, 1150, 1200, and 1250 °C.

XRD analysis showed that, for all composites, HA decomposes partially or fully into tricalcium phosphate (Ca_3_(PO_4_)_2_, TCP) during sintering. A higher temperature of sintering (*T*_s_ = 1250 °C) and a higher percentage of BG (10%) contribute to this process, but differently for composites prepared from HAP or HAG. The X-ray diffractograms for HAG composites with 5% BG demonstrate peaks for both HA and TCP for all four sintering temperatures of 1100 1150 1200, and 1250 °C (Figure 1a). For HAP composites with the same 5% BG, peaks for HA and TCP are present for three of four sintering temperatures, except *T*_s_ = 1250 °C, for which only the TCP phase was measured, indicating that HA fully decomposes into TCP during sintering (Figure 1b).

**Figure 2:**
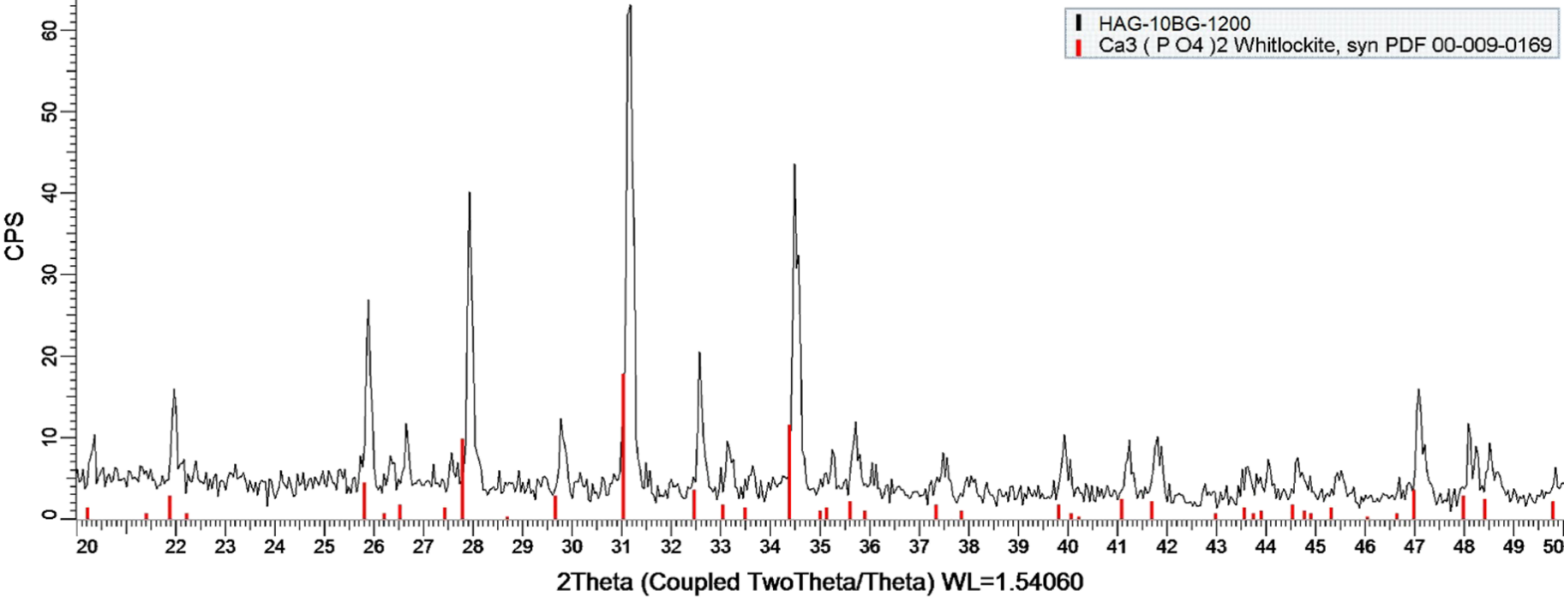
XRD pattern of the HAG-10BG-1200 composite.

The composites with 10% of bioglass exhibit the full decomposition of HA into TCP regardless of the sintering temperature (1200 or 1250 °C) and type of HA used (HAP or HAG). As an example, Figure 2 shows the XRD pattern for the HAG-10BG-1200 composite. All other patterns for composites with 10% BG, not shown here, similarly indicate the presence of solely TCP.

The increased tendency of HA to decompose into TCP with increasing sintering temperature was also observed by other authors for HA composites with B_2_O_3_ glass [30]. In this work, we observed this tendency for HA composites with a more complex glass composition.Moreover, the obtained results demonstrated that among the composites with 5% of BG the HAG-based ones are more stable against increasing sintering temperatures in comparison with HAP-based ones. The HA phase is maintained for HAG-5BG-1250, in contrast to HAP-5BG-1250, which contains only TCP phase.

The increase of glass content from 5 to 10% is also critical regarding the decomposition of HA. All composites with 10% of BG contain only TCP. Some authors observed the same effect of increasing glass content for HA composites with B_2_O_3_ glass [30] whereas other authors did not trace any influence of the glass content on the HA/TCP ratio for HA composites with 30P_2_O_5_-30CaO-40Na_2_O (mol %) glass [25]. These results, along with the ones obtained in the present work, let us to assume that a higher percentage of P_2_O_5_ (in our case, it is 40 mol %) and the addition of B_2_O_3_ (10 mol %) contribute to a stronger decomposition of HA into TCP.

The average size of the crystallites (*D_hkl_*) and the crystallinity fraction (*X*_c_) are presented in Table 3. The obtained data can be summarized as follows: (i) The average size of the crystallites proves the nanocrystalline structure of the composites, (ii) the crystallinity fraction is somewhat higher for HAP composites (HAPCs) than for HAG composites (HAGCs), and it drops for both HAPCs and HAGCs sintered at 1250 °C, and (iii) the average size of the crystallites is slightly larger for HAGCs than for HAPCs and for TCP than for HA.

**Table 3:** Average size of the crystallites and crystallinity fraction of composites.

Sample	Average size of the crystallite (*D_hkl_*), nm		Crystallinity fraction (*X*_c_), %

	TCP = Ca_3_(PO_4_)_2_	HA = Ca_5_(PO_4_)_3_(OH)	

HAP-5BG-1100	71.4	71.0	84.8
HAP-5BG-1150	70.4	68.0	79.0
HAP-5BG-1200	64.6	65.7	82.6
HAP-5BG-1250	78.6	—	75.0
HAG-5BG-1100	80.7	57.1	77.5
HAG-5BG-1150	68.3	78.4	78.7
HAG-5BG-1200	93.7	93.8	77.7
HAG-5BG-1250	63.2	49.6	74.0

### SEM investigations

**Figure 3:**
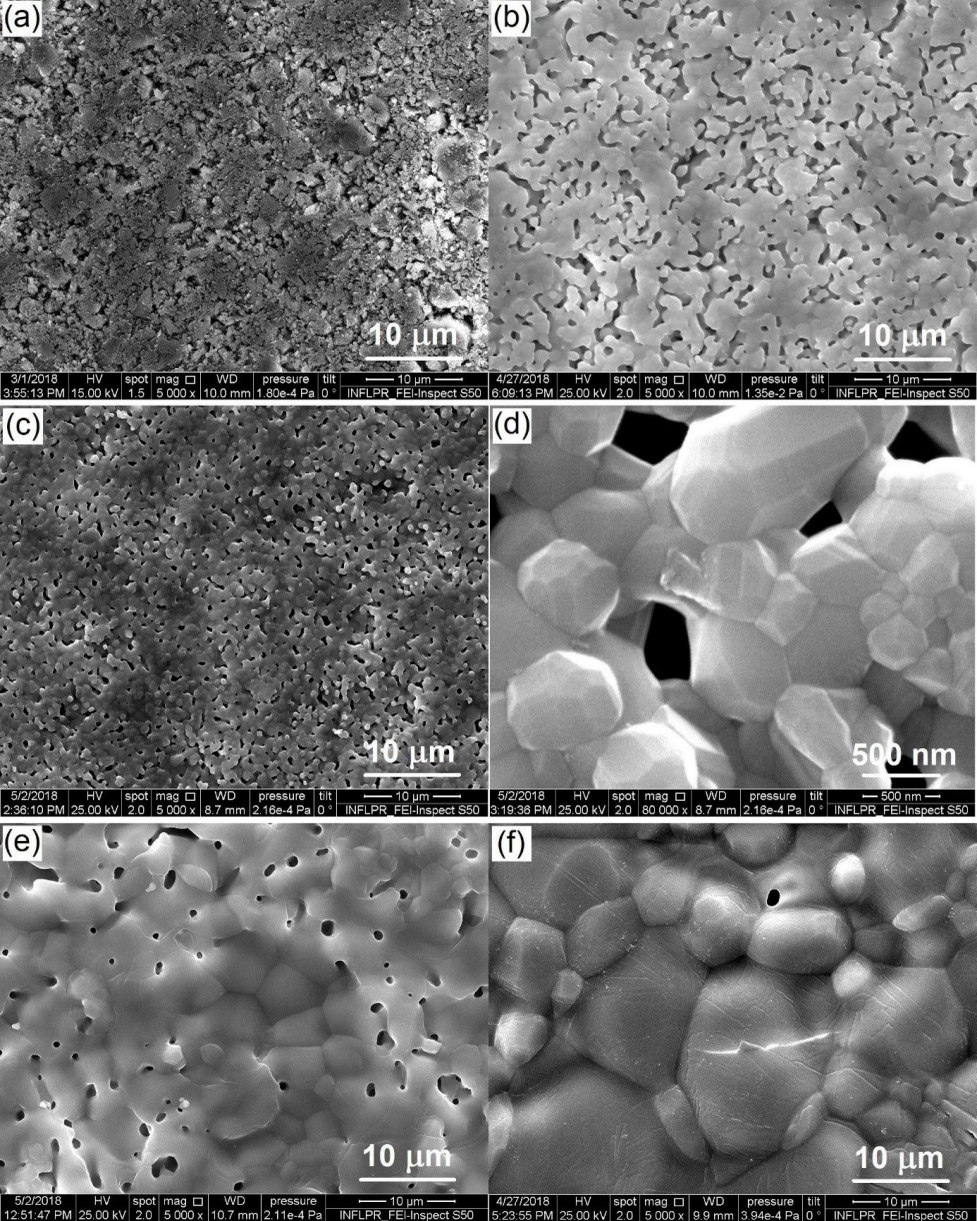
SEM images of composites. (a) HAG-5BG without sintering, (b) HAG-5BG-1100, (c, d) HAP-5BG-1100, (e) HAG-5BG-1250, and (f) HAP-5BG-1250.

Figure 3 presents SEM images showing the microstructure of the composites. After the pressing stage, the composites had a rather crumbly and porous structure (Figure 3a). Sintering at high temperatures induced fritting and compaction of the structure, which results in the reduction of porosity. This process is more pronounced with the increase of the *T*_s_ from 1100 to 1250 °C (Figure 3b vs Figure 3e and Figure 3c vs Figure 3f).

The high sintering temperatures contribute to the crystallization of HA. This is clearly seen in Figure 3d, which demonstrates the crystal faces on the HA conglomerates. If one compares the HAP- and HAG-based composites, one can see that HAPCs have a higher compaction capability than HAGCs (compare Figure 3b with Figure 3c and Figure 3e with Figure 3f).

However, the sintering temperatures of 1100 and 1150 °C appeared to be too low to create a sufficiently tough structure. Both HAP- and HAG-based composites sintered at these temperatures exhibited low bonding between constituent particles. This was manifested by the impossibility to obtain a sufficiently smooth surface during polishing, which is necessary for microindentation testing. Constituent particles were pulled out from the surface instead of being polishing. Therefore, the composites sintered at 1100 and 1150 °C were excluded from further investigations.

### Porosity and density measurements

**Figure 4:**
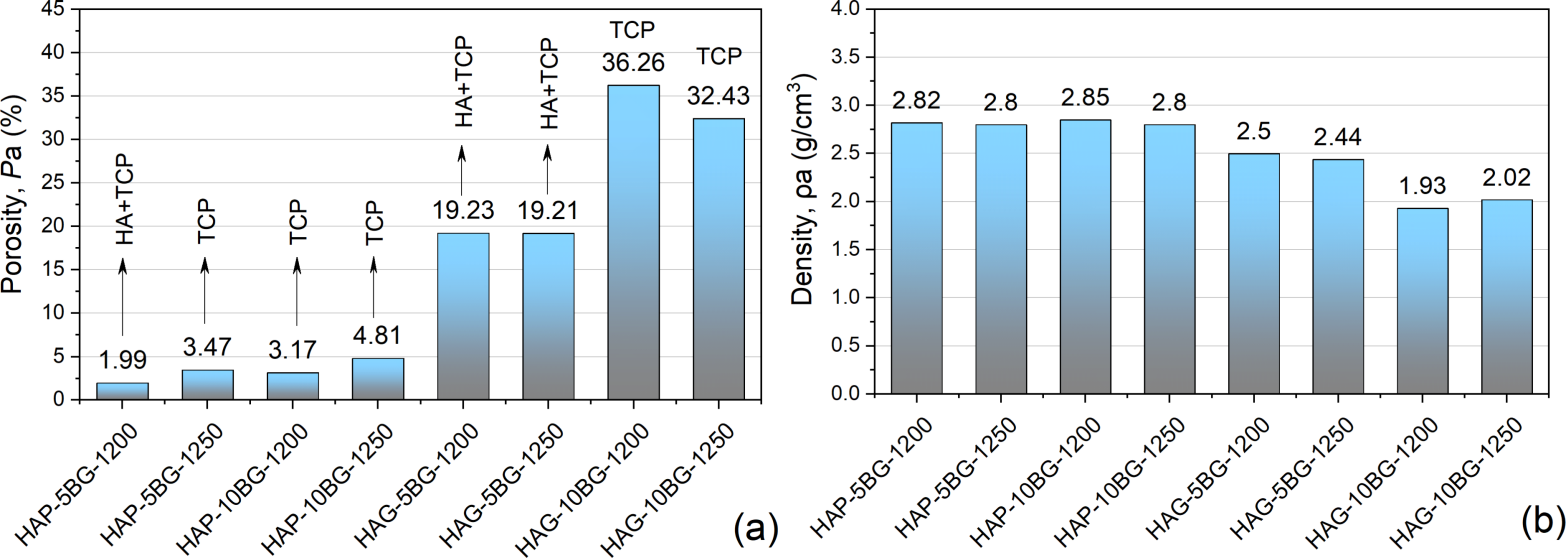
(a) Porosity in correlation with phase composition and (b) density of HAP- and HAG-based composites.

The SEM results correlate well with the density (ρ_a_) and porosity (*P*_a_) measurements of the composites, the data of which are presented in the diagrams of Figure 4. The HAGCs have significantly higher values of porosity (Figure 4a) and, correspondingly, a lower density (Figure 4b) than the HAPCs. This shows the higher compaction capability of HAPCs, confirming the SEM results.

The increase of the glass content leads to an increase of porosity, as a result of a stronger decomposition of HA into TCP. As revealed by XRD investigations and summarized in Figure 4a, for all composites, the increase of glass content from 5% to 10% leads to a transition from partial decomposition to full decomposition of HA into TCP. An exception are HAPCs sintered at 1250 °C, for which the full decomposition of HA into TCP occurs regardless of glass content. The increase of *P*_a_ with BG content is more pronounced for HAG-based composites, probably because of lower the compaction capability of the HAG particles.

The increase of the sintering temperature has different impacts on HAP- and HAG-based composites. For HAGCs, the increase of *T*_s_ induces a decrease of porosity, especially for composites with 10% of bioglass, which can result from the devitrification and shrinkage of the glass and the additional chemical bonding between the phosphate glass and TCP. In contrast, for HAPCs the increase of *T*_s_ induces an increase of porosity. For HAPCs with 5% of glass, it can be explained by the full decomposition of HA into TCP during sintering at 1250 °C, and TCP is known to be a poorly sinterable phase, hindering the densification process [41–42]. For HAPCs with 10% of glass, for which the full decomposition of HA into TCP takes place at both 1200 and 1250 °C, the increase of *P*_a_ with *T_s_* may have two reasons, namely (i) the transformation from β-TCP to α-TCP [21,43] and (ii) a more compacted structure of HAPCs and the appearance of cracks, clearly seen on SEM images (Figure 3 f), due to the non-uniform thermal expansion during the sintering at higher temperature.

### Vickers microhardness and deformation peculiarities

**Figure 5:**
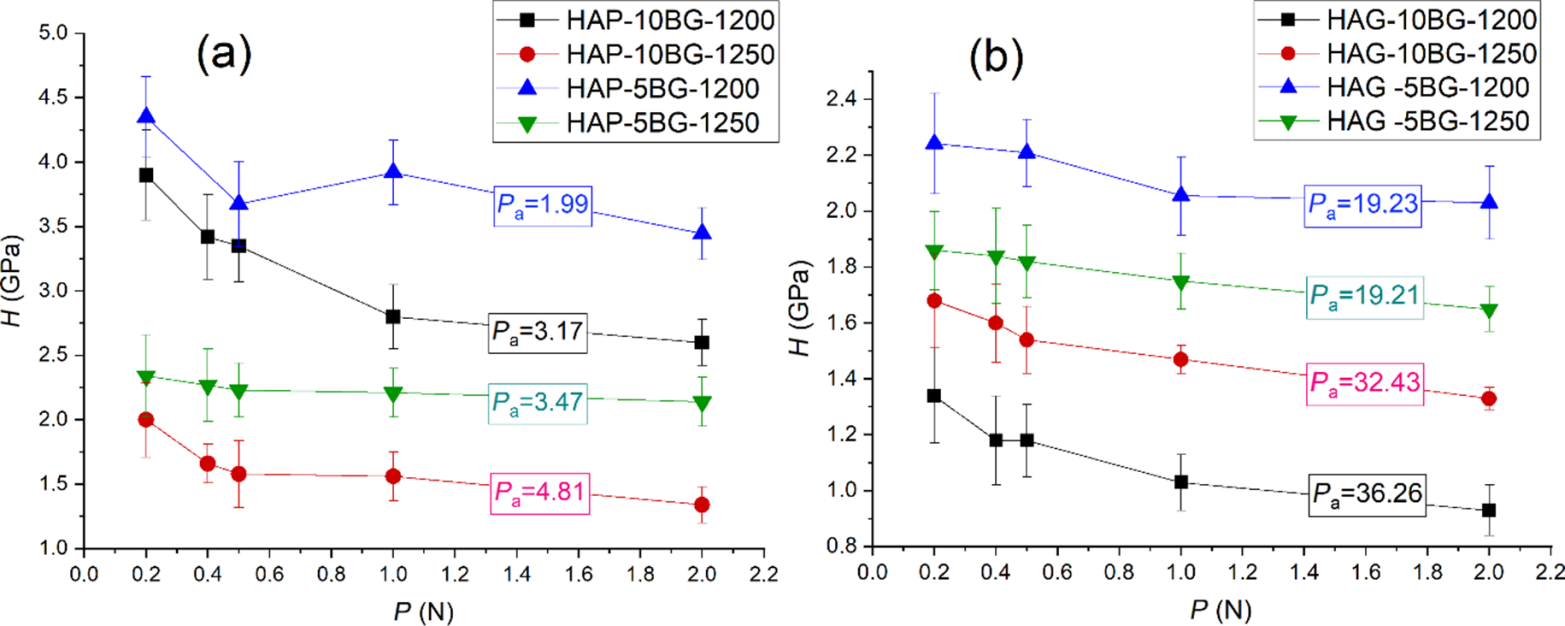
Microhardness (*H*) of (a) HAP-based composites and (b) HAG-based composites. The labels on each curve indicate the values of porosity (*P*_a_, %) for the respective composite.

Figure 5 shows the microhardness (*H*) as function of the load (*P*) applied to the Vickers indenter for HAP- and HAG-based composites. The obtained results demonstrate that the microhardness has a strong correlation with porosity (Figure 4a), namely the higher the porosity, the lower the hardness (Figure 5). This is consistent with the results obtained in other studies for hydroxyapatite [44–45] and β-TCP [46] bioceramics, which were explained on the base of the “minimum solid area” model [47]. The minimum solid area or minimum bond area between particles is the area that undergoes the maximum stresses in the porous material, therefore, the cracks spread mostly through these regions. But another important factor are the pores themselves, which represent an additional free surface facilitating plastic deformation. The material is easier to displace toward this free surface with the possibility to fill the pores, which leads to the compaction of the structure. The densification of the porous structure under Vickers indentation was demonstrated for (Bi,Pb)2Sr2Ca2Cu3Ox ceramics [48]. Thus, the higher porosity may contribute, on the one hand, to the fracture of the material and, on the other hand, to additional plastic (irreversible) deformation. Both processes lead to a decrease of hardness.

The obtained results revealed that the three modifying factors of composites, type of HA, glass content, and sintering temperature, had the following influence on the hardness (*H*) in conjunction with the porosity (*P*_a_). HAPCs, having a lower *P*_a_, exhibit higher values of *H* (Figure 5a) than HAGCs (Figure 5b). Regarding the glass content, both HAP- and HAG-based composites with 5% of BG have a higher hardness then composites with 10% of BG sintered at the same temperature. Concerning the sintering temperature, a strong correlation with *P*_a_ is observed for almost all composites and, thus, composites sintered at 1250 °C, which show higher porosity, have lower hardness (Figure 4a and Figure 5). An exception are HAG-based composites with 5% BG, which demonstrate very close values of *P*_a_ but have different values of *H*, with the higher one for HAG-5BG-1200. This result can be explained from the following considerations. The increase of the sintering temperature leads to two processes with opposite effects on the porosity/density: (i) the devitrification and shrinkage of the glass, which contribute to the densification process and (ii) the HA→β-TCP→α-TCP transformations, which increase the porosity. In the case of 5% BG, the influence of the devitrification of glass is lower than in the case of 10% BG, and it is compensated equally by the formation of TCP for sintering at 1200 and 1250 °C. Therefore, the porosity remains almost the same for both values of *T*_s_. Nevertheless, TCP affects the mechanical strength of the material resulting in the decrease of hardness with the increase of *T*_s_.

**Figure 6:**
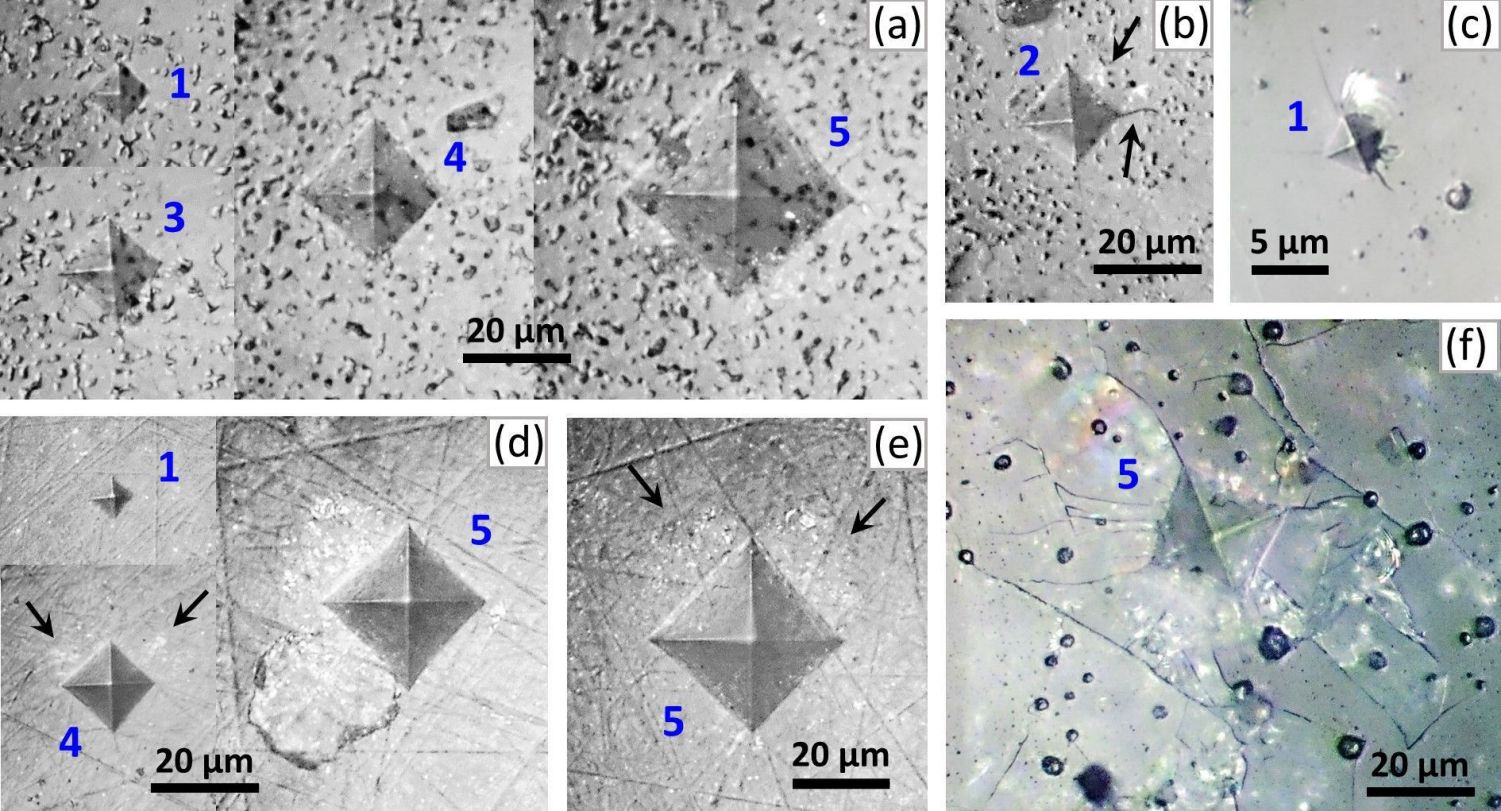
Morphology of the indentations made on (a) HAG-5BG-1250, (b) HAP-5BG-1250, (c, f) HAP-10BG-1200, (d) HAP-5BG-1200, and (e) HAG-5BG-1200. The numbers next to the indentations indicate the load (*P*) applied to the indenter: **1** = 0.2 N, **2** = 0.4 N, **3** = 0.5 N, **4** = 1.0 N, and **5** = 2.0 N. The arrows show slight delaminations displayed as fine highlights in panels (b), d), and (e), as well as a small crack in panel (b).

The analysis of the indentation morphology allowed us to differentiate three types of specific mechanical behavior of the studied composites. “Type I” is plastic behavior with no visible cracks or delaminations around the indentations (Figure 6a) or very small ones (Figure 6b). “Type II” is plastic-fragile behavior with light delaminations for loads of 0.2–1.0 N, displayed as fine highlights around the indentation, and pronounced delaminations for a load of 2.0 N (Figure 6 d,e). Note that both light and pronounced delaminations do not appear in all indentations and exhibit event frequency values from 1/10 to 5/10 for loads from 0.2 to 2.0 N, respectively. Type III is fragile behavior with pronounced cracks and delaminations beginning at the smallest load of 0.2 N (Figure 6c) and further developing for higher loads (Figure 6f). The type I is characteristic for HAG-5BG-1250, HAP-5BG-1250, HAG-10BG-1200, and HAG-10BG-1250, the type II is characteristic for HAP-5BG-1200 and HAG-5BG-1200, and the type III is characteristic for HAP-10BG-1200 and HAP-10BG-1250. Thus, HAGCs and HAPCs with 10% BG behave contrarily. The former, having the highest values of *P*_a_ (36.26% and 32.43%) deform plastically whereas the latter, having much lower values of *P*_a_ (3.17% and 4.81%) are fragile. At the same time, HAGCs and HAPCs with 5% BG behave similarly for the same values of *T*_s_ in spite of different *P*_a_ values. So HAGCs and HAPCs with 5% BG sintered at 1200 °C (*P*_a_ of 19.23% and 1.99%, respectively) show plastic-fragile behavior whereas those sintered at 1250 °C (*P*_a_ of 19.21% and 3.47 %, respectively) deform plastically. Hence, although the hardness shows a strong correlation with the porosity, one can hardly trace a strong correlation between type of mechanical behavior and porosity alone. Most probably, plastic or fragile behavior is influenced, besides the porosity, by other structural parameters such as crystallinity fraction, form and size of constituent particles, and their bonding strength. For a deeper understanding of the influence of these parameters on the plastic or fragile behavior of the composites, further investigations are necessary.

The indentation size effect (ISE) representing the increase of *H* with the decrease of *P* observed on other porous ceramics [49–51] manifests also for all composites in the present work (Figure 5). The ISE can be connected with the general deformation peculiarities of porous ceramics mentioned at the beginning of this paragraph as well as with the deformation behavior of the investigated composites. For plastically deformed composites, for which the pores may facilitate plastic deformation, the ISE may be explained as follows: Due to the smaller volume fraction of the pores with respect to the dense matrix, the probability of pores falling into the indentation zone is reduced with decreasing load. Hence, less volume of pores and more volume of dense matrix is involved in the deformation process with decreasing load, resulting in the increase of hardness. For composites with plastic-fragile and fragile behavior, the ISE is caused by involving and developing delamination and cracking with increasing load (Figure 6), which leads to the relaxation of stress in the deformation zone and, as a result, to the decrease of hardness.

### SBF testing and bioactivity of composites

**Figure 7:**
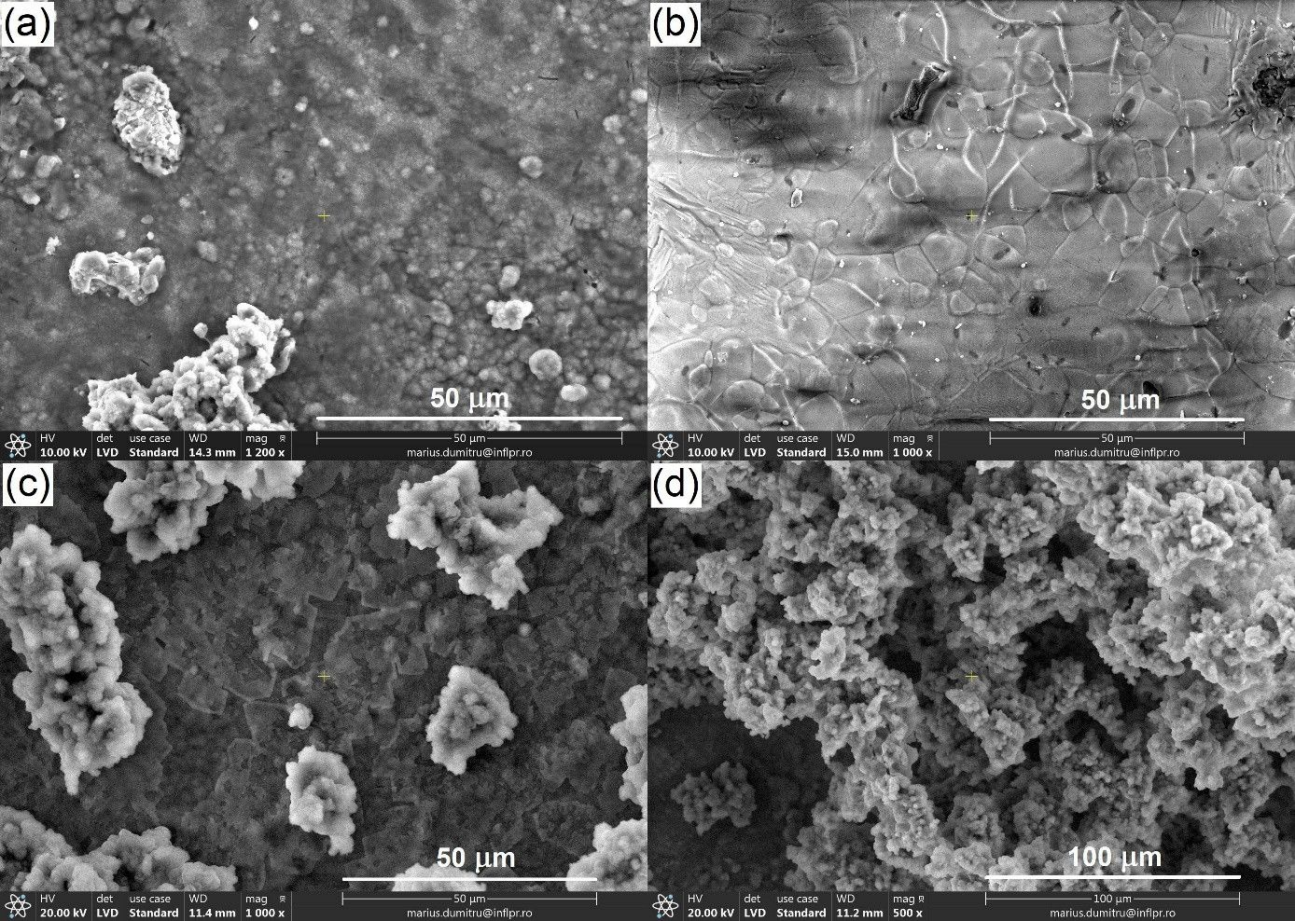
SEM images of the surface of composites after soaking in SBF. (a) HAG-5BG-1250 after 3 days, (b) HAP-5BG-1250 after 3 days, (c) HAP-5BG-1200 after 5 days, and (d) HAP-5BG-1200 after 10 days.

To assess the bioactivity of the composites they were soaked in SBF for 3, 5, 10, 15, and 30 days, after which the surface of the samples was investigated by means of SEM. The obtained SEM images led to the conclusion that the HAG-based composites had a quicker response to SBF, showing the incipient stage of calcium phosphate precipitation after 3 days of soaking (Figure 7a). In contrast, HAP-based composites did not exhibit any precipitate at the surface after 3 days of soaking (Figure 7b). This can be the result of the more porous structure of HAG-based composites (Figure 4a) and, therefore, higher dissolution rate. HAP-based composites with a denser structure need a longer period of immersion in SBF for solubilization and the beginning of the mineralization process. And indeed, after 5 days of soaking, a well-pronounced precipitate was observed on HAP-based composites, too (Figure 7c). With the increase of soaking time, the amount and thickness of the precipitate layer increased (Figure 7d) for all composites. After 15 and 30 days, the samples were entirely covered with a layer of apatite precipitate of about 2–3 µm thickness with no difference between HAP- and HAG-based composites. We did not observe any influence of the glass content (5% or 10%) or sintering temperature (1200 or 1250 °C) on the mineralization capability of the composites.

**Figure 8:**
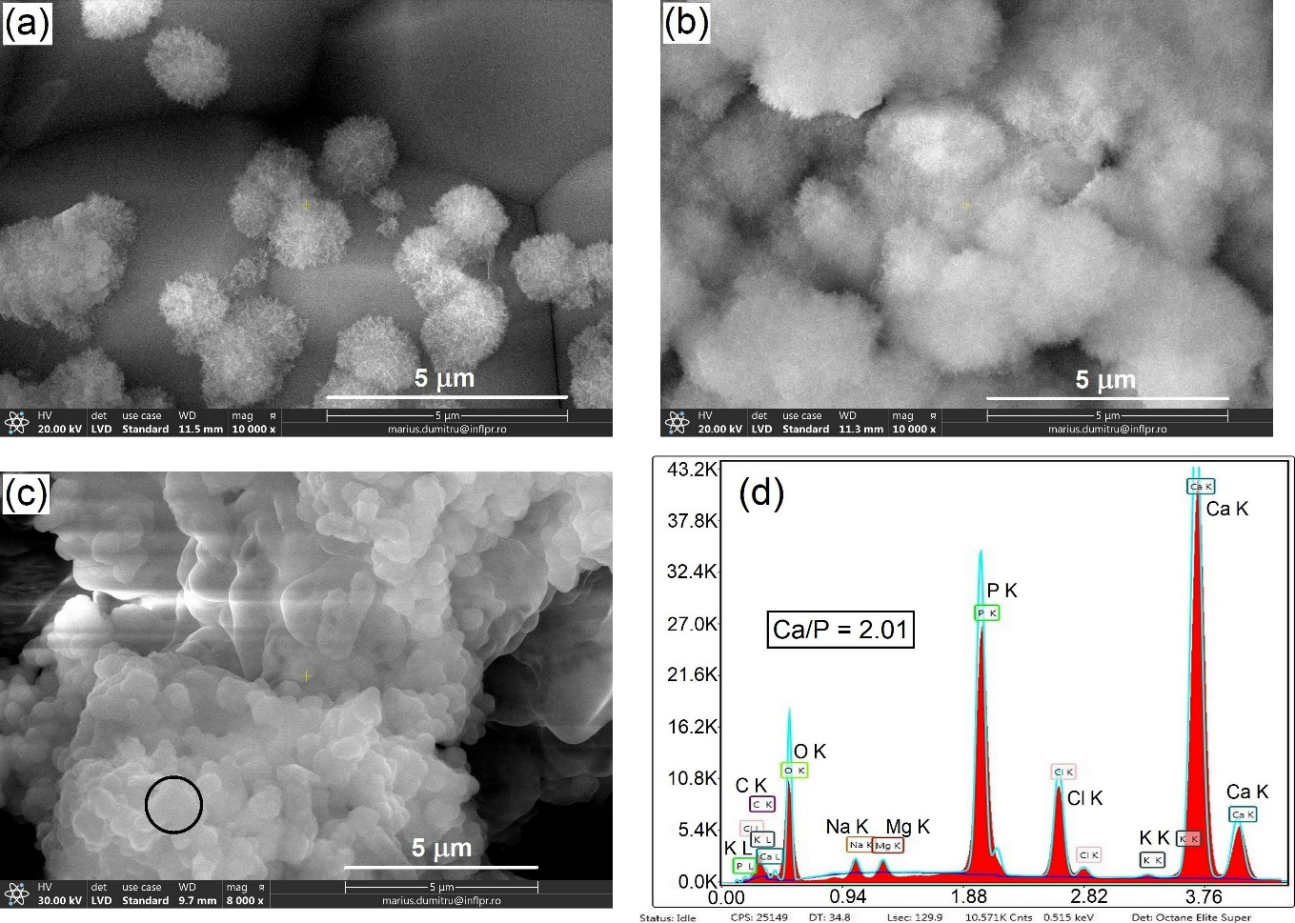
SEM images of the HAP-5BG-1200 surface after soaking in SBF solution for (a) 5 days, (b) 10 days, and (c) 15 days. (d) EDX spectrum from the region indicated by the circle in panel (c).

At higher magnification, the evolution of the fine microstructure of the calcium phosphate precipitates with soaking time can be traced (Figure 8). One can see that, at the incipient stage, the precipitate grows in the form of separate needle-shaped airy globules of about 1 µm diameter (Figure 8a). Similar needle-like particles were observed by Hyun-Min Kim et al. in TEM investigations after SBF immersion of synthetic hydroxyapatite and were proven to be apatite crystals grown together [52]. With the increase of soaking time, the amount and dimensions of these globules increased, forming a continuous cotton-like fluffy layer (Figure 8b). When the immersion time exceeded 10 days, a denser druse-like structure was formed (Figure 8c).

The EDX analysis demonstrated that the precipitate layer contained Ca, P, Na, Mg, and K, with highest fractions of Ca and P (Figure 8d). The Ca/P ratio takes values between 1.23 and 2.33 with generally lower values for HAG-based composites and the tendency to increase with soaking time in SBF for both types of composites (HAGCs and HAPCs). The Ca/P ratio of stoichiometric hydroxyapatite is 1.67. Lower values of Ca/P ratio at the early stages of biomineralization are in agreement with the results obtained in other works for Bioglass^®^-type glasses, hydroxyapatite, and A-W glass ceramics [7,52–55] and can be explained by the formation of CaO-P_2_O_5_ or other calcium deficient phases. At the later stages of biomineralization, that is, after longer soaking times, the precipitate undergoes structural and chemical transformation resulting in the formation of carbonate-hydroxyapatite (CHA). The obtained values of Ca/P ratio higher than 1.67 suggest the formation of B-type CHA, characteristic for biological hydroxyapatite, in which the PO_4_^3−^ ions are substituted by CO_3_^2−^ ions, accordingly increasing the Ca/P ratio [56]. This is confirmed by the EDX analysis showing the presence of C (Figure 8d).

The pH value of the initial SBF was 7.33–7.34, in agreement with the necessary conditions for SBF bioactivity testing [23,25,36,40]. Submersing the composites in SBF increased its pH to of 7.89–7.94 after 3 days of soaking, which remained constant after 5, 10, 15, and 30 days. No influence of the type of hydroxyapatite (HAP or HAG), glass content (5 or 10%), or sintering temperature (1200 or 1250°C) on the pH value of SBF was observed. The change of the surrounding physiologic environment to alkaline conditions was caused by the dissolution of the composite in SBF, and it is considered to be favorable for bone cell proliferation, although a high pH value may be detrimental to optimal osteoblast metabolism [14]. It was shown, for example, that values of pH 7.0–7.5 were optimal for osteoclast differentiation and proliferation [57].

### MTT assay and biocompatibility of composites

**Figure 9:**
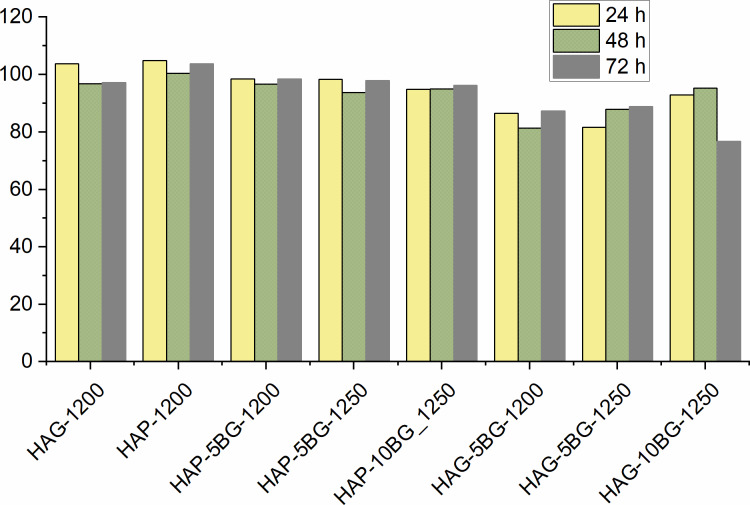
Relative viability of bone marrow STEM cells exposed to HAP- and HAG-based composites after 24, 48, and 72 h of cell culture.

Figure 9 shows the results of a preliminary biological study performed by MTT assay regarding the relative viability of the bone marrow STEM cells exposed to HAP- and HAG-based composites. As a control, HAP and HAG ceramics sintered at 1200 °C without glass addition (labeled as HAP-1200 and HAG-1200, respectively) were included in the test. One can see that HAP-based composites demonstrate a good biocompatibility with relative cell viability values between 94% and 99%, very closed to those for the HAP-1200 and HAG-1200 control samples. A somewhat lower biocompatibility is shown by HAG-based composites. It is most probably caused by the more porous structure and, as a result, higher dissolution rate of these composites, inducing an increased ion concentration in the surrounding biological environment, which may be detrimental for cell proliferation. It can be noticed that HAG-1200 ceramics without BG also exhibited a somewhat lower cell viability than HAP-1200 ceramics. For both HAP- and HAG-based composites the addition of 10% of BG decreased the values of relative viability, again due to higher ion concentration in the physiologic environment due to the higher porosity and dissolubility.

## Conclusion

A series of nanocomposites with two types of hydroxyapatite, obtained by precipitate (HAP) or sol–gel (HAG) methods, and a boro-silico-phosphate glass in their composition were synthesized and studied. The influence of the type of hydroxyapatite (HAP and HAG), glass content (5% and 10%), and sintering temperature (1100–1250 °C) on microstructure, chemical, mechanical, and biological properties were investigated. The following results were obtained: (i) The rather high percentage of P_2_O_5_ (40 mol %) and the addition of B_2_O_3_ (10 mol %) in the glass content contributed to a more intensive decomposition of the HA into TCP. The same influence was observed for the highest sintering temperature (*T*_s_* =* 1250 °C) and the highest percentage of BG (10%). HAG-based composites with 5% BG were found to be more stable against increasing sintering temperatures. (ii) HAP showed higher compaction capability than HAG and, as a result, the porosity *P*_a_ of HAP-based composites (HAPCs) is significantly lower than that of HAG-based composites (HAGCs). The increase of the glass content leads to the increase of *P*_a_. The increase of *T*_s_ raises the *P*_a_ of HAPCs and lowers the *P*_a_ of HAGCs. These peculiarities are assumed to be connected with different contributions of processes such as devitrification of glass and HA→TCP transformation during sintering. (iii) The microhardness *H* of the composites showed a strong correlation with porosity, that is, the higher the porosity, the lower the hardness. Thus, the values of *H* are higher for HAPCs than for HAGCs, and they decrease with the increase of glass content and sintering temperature. (iv) HAGCs showed a quicker response to SBF within the first days of soaking. However, after longer soaking times, no difference was observed between HAGCs and HAPCs, and both groups of composites showed good mineralization regardless of sintering temperature and glass content. The evolution of the fine microstructure of the calcium phosphate precipitate with soaking time was traced. (v) A biocompatibility with relative cell viability values between 94% and 99% was revealed by MTT assay for HAPCs. Slightly lower values were observed for HAGCs, caused by the more porous structure and the higher dissolution rate inducing an increased ion concentration in the surrounding biological environment.

In the range of the examined composites, an optimal combination of properties (structural, mechanical, chemical and biological) was found in the HAP-based composite with 5% of bioglass sintered at 1200 °C (HAP-5BG-1200). This sample contains a beneficial combination of HA and TCP and has the lowest porosity (*P*_a_ = 1.99), the highest microhardness (*H* = 3.45–4.35 GPa), and, at the same time, a good bioactivity (solubility and biomineralization) and biocompatibility (98.5% of cell viability).

## Annex

### Ethical approval and the protocol used for the isolation of the mesenchymal stem cells

The mesenchymal stem cells (MSC) for the MTT test were isolated from the bone marrow of six-months-old Wistar rats at the Laboratory of Tissue Engineering and Cells Culture of the Nicolae Testemitanu State University of Medicine and Pharmacy (NTSUMP) of the Republic of Moldova. The laboratory has the permission from the University Research Ethics Committee acting in accordance with the Statute of the Research Ethics Committee of NTSUMP to extract and perform experiments with Wistar rat organs, tissues, and cells (Ethics Committee Approval of NTSUMP No 41 from 03.02.2020). The protocol used for the isolation and identification of the mesenchymal stem cells is described below.

The bone marrow extracted from the medullary canal and metaphyseal regions of the Wistar rat bones was washed with a 2 mL syringe with PBS (HI Media, India) into a 50 mL centrifuge tube. The tube was centrifuged at 170*g* for 10 min. After removing the supernatant, the centrifuged cells were resuspended in 10 mL of HiMesoXL™ mesenchymal stem cell expansion medium (HiMedia, India) and pipetted several times to generate a single-cell suspension. After repeated centrifugation, the cells were resuspended in mesenchymal stem cell expansion medium and transferred into a 25 cm^2^ cell culture flask and incubated at 37 °C in 5%CO_2_ humid environment. In the first passage, only half of the volume of MSC expansion medium was changed until 40–50% cellular confluence, with the subsequent change of all medium. The medium was changed every 2 days, except for the first change, which was made on the third day. At 70–80% cellular confluence, the cells were detached with a small quantity of 0.25% trypsin/0.02% EDTA. The cells were transferred in a 75 cm^2^ flask and cultured in mesenchymal stem cell expansion medium under the same conditions, changing the cell culture medium every second day, until 70–90% cellular confluence. After detachment, the cells were split and cultured in the third passage in three 75cm^2^ cell culture flasks under the same conditions. The cells were stored at 1·10^6^ cells/cryovial at −80°C in FBS (Lonza, Belgium) with 10%DMSO (Alchimia, Italy).

To obtain a pure mesenchymal stem cell culture, the bone marrow cells were cultured specifically in commercially available HiMesoXL™ mesenchymal stem cell expansion medium to isolate only mesenchymal stem cells. Also, to remove contaminating cells, the isolated cells were cultured over three passages. Mesenchymal stem cells isolated in the same way were used as control in the identification of bone cells; all cell groups were cultured in over-confluence for 18 days [58]. After 18 days of culture in over-confluence, the mesenchymal stem cell control did not form the mineralized nodules that could have been stained with alizarin Red.
